# The Differentiation and Protective Function of Cytolytic CD4 T Cells in Influenza Infection

**DOI:** 10.3389/fimmu.2016.00093

**Published:** 2016-03-09

**Authors:** Deborah M. Brown, Anna T. Lampe, Aspen M. Workman

**Affiliations:** ^1^School of Biological Sciences, University of Nebraska-Lincoln, Lincoln, NE, USA; ^2^Nebraska Center for Virology, University of Nebraska-Lincoln, Lincoln, NE, USA

**Keywords:** CD4 effectors, CD4 cytolytic cells, influenza A virus, perforin, granzyme B

## Abstract

CD4 T cells that recognize peptide antigen in the context of class II MHC can differentiate into various subsets that are characterized by their helper functions. However, increasing evidence indicates that CD4 cells with direct cytolytic activity (CD4 CTL) play a role in chronic as well as acute infections, such as influenza A virus (IAV) infection. In the last couple of decades, techniques to measure the frequency and activity of these cytolytic cells has demonstrated their abundance in infections, such as human immunodeficiency virus, mouse pox, murine gamma herpes virus, cytomegalovirus, Epstein–Barr virus, and influenza among others. We now appreciate a greater role for CD4 CTL as direct effectors in viral infections and antitumor immunity through their ability to acquire perforin-mediated cytolytic activity and contribution to lysis of virally infected targets or tumors. As early as the 1980s, CD4 T cell clones with cytolytic potential were identified after influenza virus infection, yet much of this early work was dependent on *in vitro* culture and little was known about the physiological relevance of CD4 CTL. Here, we discuss the direct role CD4 CTL play in protection against lethal IAV infection and the factors that drive the generation of perforin-mediated lytic activity in CD4 cells *in vivo* during IAV infection. While focusing on CD4 CTL generated during IAV infection, we pull comparisons from the literature in other antiviral and antitumor systems. Further, we highlight what is currently known about CD4 CTL secondary and memory responses, as well as vaccination strategies to induce these potent killer cells that provide an extra layer of cell-mediated immune protection against heterosubtypic IAV infection.

## CD4 CTL: Historical Perspective

CD4 T cells differentiate into a number of different subsets based on the cytokine milieu and transcription factors expressed during activation and differentiation. These subsets are identified by canonical cytokine secretion patterns and their effector function. Traditional helper cell functions during infection include TNF-α and IFN-γ secretion that activates other immune cells and can protect against intracellular infections (Th1), IL-4-induced class switching to IgE, and protection against extracellular worm infections (Th2), IL-17, and IL-22 secretion that promotes protection against extracellular bacterial and fungal infections (Th17), and IL-21 secretion that facilitates germinal center formation and class switching to protein antigens (Tfh) (1, 2). There has recently been an increase in the literature surrounding the differentiation, function, and memory potential of class II-restricted CD4 T cells with cytolytic properties (termed CD4 CTL), yet identification of these cells dates back almost 40 years. Using Lyt-1^+^ (CD4) T cell clones, it was demonstrated that “helper” T cells could lyse allogeneic target cells similar to Lyt-2^+^ (CD8) cells (3, 4). Although early work utilized allogeneic CD4 cell clones due to their high frequency in normal mice, in 1985, CD4^+^ T cell clones were isolated after influenza A/Jap/57 infection that were able to lyse influenza-infected target cells expressing class II, but not class I MHC, suggesting that CD4 CTL activity developed after infection and subsequent *in vitro* culture (5). Further experiments determined that soluble mediators were not responsible for conferring cytotoxicity, suggesting direct cell contact was required for lysis. Although these clones could also promote differing levels of helper activity in *in vitro* cultures, the magnitude of B cell antibody production was inversely correlated to cytotoxic activity (5). Although CD4 CTL could be generated *in vitro*, the identification and subsequent *in vivo* relevance of these cells was not appreciated until years later.

In the last 10–15 years, accumulating evidence that CD4 CTL are not an artifact of *in vitro* stimulation but rather develop *via* an alternative activation pathway separate from canonical T helper cells has gained acceptance. Most of this literature focused on the appearance or activation of CD4 CTL during chronic infection suggesting that repeated antigen exposure and terminal differentiation were hallmarks of the CD4 CTL phenotype (6, 7). CD4 CTL developed in response to cytomegalovirus (CMV) (7, 8), human immunodeficiency virus (HIV) (9, 10), and Epstein–Barr virus (EBV) (11, 12) infection in humans as well as lymphocytic choriomeningitis virus (LCMV) (13, 14) and mouse gammaherpes virus (15, 16) in mice. In most of these infections, CD4 CTL were important effector cells and, in some cases, responsible for vaccination-induced protection against infection (17, 18). However, CD4 CTL have also been identified in acute infections, such as LCMV clone 13 (13), mouse pox (17), and influenza A virus (IAV) infection in both humans (19) and mice (20–22).

Many of the early reports detailing the appearance of CD4 CTL did not identify the mechanism of cell killing by clones generated *in vitro* although, by the 1990s, it was reported that the cell surface receptor:ligand pair, Fas:FasL, was a major mechanism of cell death induced by CD4 CTL during interaction with virally infected B cells (23) or in infection with LCMV (14). More recent data have provided evidence that CD4 CTL can utilize the perforin–granzyme pathway of target cell killing to act as primary lytic effectors for many viral infections (24) including mouse gamma herpes virus (15), mouse pox (17), and IAV (19–21). In fact, during IAV infection *in vivo*, CD4 CTL exclusively use the perforin–granzyme B (GrB) pathway for cell killing.

## *In Vitro* and *In Vivo* Generated CD4 CTL as Direct Effectors against Influenza Infection

Building on early evidence that CD4 CTL clones could be generated by IAV infection (5), Graham and Braciale generated a panel of CD4 Th1 and Th2 helper cell clones after inactivated IAV and incomplete Freund’s adjuvant injection (25). It was shown that Th1 clones, but not Th2 clones, could lyse influenza infected targets and indicated that CD4 CTL could be generated after vaccination as well as live infection. Further, Th1 clones, but not Th2 clones, could protect mice against lethal influenza infection, although the mechanism of killing was not elucidated at that time (25). With the advent of TCR tg mice specific for influenza peptide from hemagglutinin (HA) 126–138 in the context of class II I-A^d^, Brown et al. revisited the idea that only Th1 polarized cells acquired cytolytic activity and investigated the mechanisms of protection in a monoclonal population of CD4 effectors generated *in vitro*. It was demonstrated that CD4^+^ effectors generated under Th1 polarizing conditions, but not Th2 polarizing conditions, could protect mice against highly pathogenic H1N1 IAV infection after adoptive transfer (26). Interestingly, CD4 T cells needed to be activated prior to adoptive transfer, as high numbers of naïve CD4 cells could not afford protection against lethal Influenza A/Puerto Rico/8/34 (PR8) infection. It was shown that host T cells were not required for protection mediated by CD4 effectors; however, host B cell antibody production was induced by Th1 effectors and this effect was IFN-γ independent. Moreover, at high dose PR8 infection (10 LD_50_), CD4 effectors and passive transfer of antibodies protected B cell-deficient mice from highly pathogenic H1N1 infection (26). Surprisingly, a more direct role for CD4 effectors was found when B cell-deficient mice were given Th1 effectors and survived a lethal dose of PR8 H1N1. Additionally, it was demonstrated that perforin-mediated cytotoxicity was the mechanism utilized by Th1 effectors responsible for protection as perforin-deficient CD4 cells could not abrogate weight loss or survival. These cytolytic CD4 effectors resembled the early anti-influenza Th1 clones generated by Graham and Braciale, yet were developed *in vitro* with peptide pulsed APC and cytokine polarization. Culture conditions for generating these potent anti-influenza effectors required low dose peptide pulsed antigen-presenting cells, as anti-CD3 and anti-CD28 or high concentrations of soluble peptide added directly to cultures of APC and T cells did not induce antigen-specific cytolytic CD4 cells (27). This further suggests that an APC-derived second signal is required to generate protective CD4 CTL.

Protection from lethal dose IAV infection mediated by Th1 clones or effectors generated *in vitro* indicated that CD4 CTL could have direct effects on viral replication. However, these *in vitro* generated effectors were quite homogeneous and displayed characteristics that might not reflect physiological phenotypes. Thus, IAV-specific CD4 effectors were generated *in vivo* by isolating CD4^+^ cells from the lung and draining lymph node (DLN) at the peak of the response after sublethal PR8 infection and adoptively transferred into naïve hosts that were then given lethal doses of PR8. These IAV-specific CD4^+^ cells protected mice from five LD_50_ dose of PR8 IAV infection, but unlike *in vitro*-generated effectors, protection mediated by *in vivo*-generated effectors was partially dependent on IFN-γ. Similar to *in vitro*-generated effectors, primary *in vivo*-generated effectors developed perforin-mediated cytotoxicity that was partly responsible for protection in a model of lethal IAV infection (20).

Investigators have also shown direct effects of memory CD4 T cells in protection against influenza infection (28, 29). These studies used an adoptive transfer system in which naïve TCR tg CD4 cells specific for HA peptide were primed *in vitro*, subsequently transferred to normal mice, and allowed to transit to the memory phase. It was demonstrated by two independent groups that CD4 memory cells had direct effects on influenza virus clearance (28, 29). Further studies using CD4 memory cells deficient in IFN-γ, perforin (Prf1), or host mice deficient in different cell populations demonstrated that CD4 memory cells protected mice using multiple and synergizing mechanisms where host immune cells, IFN-γ, and perforin all played a role (28, 30). Viral replication was blunted by both WT and Prf1^−/−^ effectors; however, a high percentage of viral escape mutants were isolated from mice given WT, but not Prf1^−/−^ memory cells, suggesting that perforin expressing CD4 CTL drive selection of epitope-specific escape mutants during lethal influenza infection. Overall, the requirement for perforin expression by CD4 CTL in promoting protection against lethal IAV infection was dependent on effector cell activation and immune competency of recipient mice. Nonetheless, CD4 CTL can mediate viral clearance directly through cytotoxic mechanisms.

## CD4 CTL in the Primary Response to Influenza

The overwhelming evidence that CD4 CTL provided protection against lethal IAV infection led us to investigate whether CD4 CTL could be activated during the primary response to IAV. Brown et al. tracked both the TCR tg CD4 response specific for influenza HA_126–138_ in an adoptive transfer model and the endogenous peptide specific response to characterize the CD4 CTL response to primary infection (20). Using CD4 TCR tg cells, it was concluded that only cells in lung, but not DLN, expressed GrB and acquired perforin-mediated cytotoxicity by 7 days post-IAV infection. Moreover, the same result in the endogenous response to infection was observed, but this response peaked at days 9–10. Not surprisingly, the GrB and perforin levels and lytic capacity of CD4 CTL in our study did not reach the magnitude as that seen in CD8^+^ CTL (20). This is in contrast to the LCMV system, in which CD4 CTL were reported to be as robust as CD8 CTL in lytic activity when adjusted for E:T ratios and functional avidities (31). Our findings were confirmed and extended by Hua et al., in which influenza-infected mice increased GrB and perforin expression by CD4 effectors in the lung, but not the DLN, 7 days post-infection. The TCR tg adoptive transfer model using Ova-specific CD4 cells (OT-II) and Ova-expressing PR8 virus was used in this report. Moreover, antigen-specific CD4^+^ T cells (as measured by IFN-γ expression) in the lung could degranulate as determined by increased CD107a expression (21). Taken together, two independent groups have determined that CD4 CTL activity is apparent in CD4 effectors in the lung, which correlates to IFN-γ expression (21), but does not require INF-γ for differentiation or effector function (20).

Unlike CD8^+^ CTL, in which a small percentage of cells express GrB in the DLN and can lyse target cells, CD4 CTL in the DLN express little to no GrB protein by flow cytometric analysis. In contrast, Hua et al. demonstrated that approximately 20% of the DLN CD4^+^ cells generated after IAV infection expressed perforin protein, yet our work suggests that CD4 CTL in secondary lymphoid organs (SLO) do not have the capacity to lyse peptide expressing targets, even when directly isolated *via* cell sorting techniques (20). While intracellular GrB expression can be detected directly *ex vivo*, perforin detection in both CD8 and CD4 cells by flow cytometry has proven more difficult (31–33). Polyclonal activation with PMA and ionomycin was necessary to detect perforin protein intracellularly in DLN CD4 cells by flow cytometry (21) yet direct *ex vivo* expression or expression after peptide restimulation has been difficult to detect (22, 31, 34). These issues need further investigation to elucidate the relationship between GrB and perforin expression in influenza infection. Analysis of perforin expression by Western blot in *in vitro*-generated CD8 (34) and CD4 (22) T cells has demonstrated that GrB and perforin are regulated differently with GrB usually expressed prior to and at higher levels than perforin. Moreover, not all GrB expressing cells can degranulate or mediate killing activity suggesting that GrB expression does not directly correlate to perforin expression (35). Recently, a new method has been described that utilizes fixatives commonly used in immunohistochemistry to demonstrate perforin expression by flow cytometry in CD8 CTL after *in vitro* activation (32). GrB and perforin double staining has been shown by this technique and our lab is pursuing this method to further understand the expression patterns of GrB and perforin and how that relates to degranulation in CD4 CTL during influenza infection.

Although work presented thus far deals exclusively with CD4 CTL in mouse models of IAV infection, there have been reports detailing the identification of CD4 CTL in humans after vaccination (36, 37) or experimental IAV infection (19). Wilkinson et al. demonstrated that influenza-specific CD4 cells expressing GrB and exhibiting CTL activity were present at baseline and 7 days post-IAV infection in healthy volunteers. Moreover, CD4-specific and perforin-dependent CTL activity correlated to lower disease metrics as measured by illness duration and symptom scores. In this study, volunteers were excluded on the basis of preexisting antibody responses to the experimental strains and CD8 T cell responses did not appear to correlate to disease scores (19). However, in a study done in populations naturally exposed to pandemic IAV, it was demonstrated that a higher frequency of cross reactive IFN-γ^+^ CD8^+^ T cells correlated to asymptomatic protection in the absence of neutralizing antibodies (38). In these correlation studies, the cooperative effects of CD4 and CD8 T cells cannot be adequately controlled for, yet both CD4 and CD8 T cells are specific for the internal, conserved IAV proteins suggesting that T cells may cooperate to diminish viral titers early in infection. In a mouse model of protection, McKinstry et al. demonstrated that CD4 memory cells were more effective when transferred to CD8-sufficient hosts compared to CD8-deficient hosts, suggesting that CD4 memory cells can cooperate with CD8 cells in promoting protection (28). Taken together, CD4 CTL represent an important antiviral effector of cell-mediated immunity that has direct effects on viral titers and disease progression in humans (19) as well as mice (20, 26, 28).

## Factors that Drive the Differentiation of CD4 CTL

Various studies have begun to elucidate the factors that drive CD4 CTL differentiation during influenza infection *in vivo* (20–22, 39). While it has become apparent that cytokines and transcription factors important in driving CD8 CTL function may have a role in CD4 CTL differentiation, these cell types remain fundamentally distinct. However, similar to CD8 CTL generation, a role for IL-2 in driving the cytolytic phenotype in CD4 T cells was identified (22, 27). *In vitro*, low dose (10 ng/ml) exogenous IL-2 promotes high levels of GrB and moderate levels of perforin as measured by Western blot (22). While these *in vitro*-generated CD4 CTL can lyse targets with high efficiency, the use of Prf1-knockout T cells indicated that the cytolytic activity that does occur is partially mediated by Fas/FasL and partially mediated by perforin (20, 27). The correlation of CTL activity and GrB expression in the Th1, but not Th2, subsets led to further investigation of the cytokine factors necessary for CD4 CTL generation *in vitro*. Although naïve cells incubated with peptide pulsed APC and IL-2 alone induced GrB and CTL activity, it was demonstrated that addition of IL-12 or IL-6 could induce higher levels of perforin expression by Western blot (40). Moreover, addition of the type I interferon (IFN), IFN-α to *in vitro* cultures results in slight increases in GrB production by CD4 T cells and IFN-α and IL-2 in combination have synergistic effects on GrB production, perforin expression, and CD4 CTL target killing (21). Interestingly, *in vivo*, IAV infection drives CTL activity mediated exclusively by perforin (19, 20), making an interpretation of the cytokine requirements for driving this cell type during natural infection relatively straightforward (21, 22, 39).

The role of IL-2 signaling in driving CD4 CTL activity *in vivo* was revealed by the use of high affinity IL-2 receptor (Il2rα)-deficient mice (21, 22). IL-2Rα deficiency resulted in low GrB expression in the lung indicating a requirement for IL-2 signaling for CD4 CTL differentiation (21, 22). Interestingly, there was a dose dependent requirement for IL-2 receptor signaling as CD4 T cells that have intermediate expression of IL-2Rα (IL-2Rα^+/−^) exhibit intermediate GrB production and target cell killing compared to wild type CD4 T cells and IL-2Rα^−/−^ CD4 T cells (22).

As discussed above, CD4 T cells expressing GrB and perforin can be found at the site of infection, the lung, but not the DLN or spleen, after IAV infection (20–22). This localized phenotype suggests that different conditions in these two environments affect the development of CD4 CTL, with the environment of the lung being better suited for CD4 CTL differentiation and activity. As the lung is the site of active viral replication, one difference between the lung environment and the DLN after IAV infection is the composition and amount of cytokines present. The lung expresses higher amounts of IFN-α than the DLN at day 5 post-infection (21) as well as other proinflammatory cytokines, such as IL-6 and TNF-α (40). The necessity for Type I IFN in promoting the cytolytic phenotype was confirmed using IFNAR1-deficient mice that were infected with IAV. Compared to WT mice, IFNAR1-deficient mice showed lower levels of GrB and perforin, and this was further decreased in the presence of anti-IL-2 antibodies, suggesting that type I IFN and IL-2 cooperate to drive CD4 CTL generation during IAV infection (21).

Cytokine signaling results in the activation of kinases and subsequent phosphorylation and dimerization of signal transducer and activator of transcription (STAT) proteins. Since it has been determined that IL-2 is required for CD4 CTL development, the role played by Jak3 and STAT5 have been investigated. Pharmacological inhibitors of both Jak3 and STAT5 lead to decreased GrB expression and lower cytolytic activity (22). Taken together, these results suggest that extended signaling through Jak3 and STAT5 induced by IL-2 are necessary for optimal CD4 CTL formation. In addition, Type I IFNs, such as IFN-α, signal through the signal transducer STAT2, which appears to play a role in GrB expression, as STAT2 deficiency results in decreased GrB expression by CD4 T cells in the lung after IAV infection (21). Investigating other intracellular signaling pathways involved in driving perforin-mediated CD4 CTL activity will provide further insight into the environmental signals required for CD4 CTL differentiation.

Whether the same antigen-presenting cell delivers these signals simultaneously and whether distinct signals are separated by space and time are questions that remain to be answered. It is interesting to postulate that migratory dendritic cells that deliver antigen to the DLN from the lung could provide initial signals for activation, such as IL-12, that induce autocrine IL-2 production by CD4 CTL precursors. Data from Brown et al. and Hua et al. support this notion as IL-2 production in the DLN is readily apparent (21), even in situations where inflammation in the lung is different (40). Once CD4 CTL precursors migrate to the lung, they receive additional signals, such as IFN-α (21) or IL-6, from alveolar Mφ or other APC in the parenchyma (20). After terminal differentiation into competent CTL, CD4 cells can migrate to the airways to interact with and lyse class II expressing epithelial cells (20), thus providing an extra layer of heterosubtypic protection, especially for highly pathogenic IAV strains.

It has been known for many years that transcription factors regulate the differentiation of distinct CD4 T helper cell subsets with T-bet being the master regulator of Th1 polarization and GATA-3 driving Th2 polarization [reviewed in Ref. (1, 2)]. However, whether CD4 CTL comprise a new type of T helper subset, with distinct transcription factor requirements, is a matter of intense investigation. In many cases, CD4 CTL differentiation follows a Th1 developmental pathway with IL-12 but not IL-4 contributing to GrB and perforin induction (26, 27). CD8 CTL have been shown to require the coordinated expression of Runx3, T-bet, and Eomesodermin (Eomes) for full CTL activity and progression to memory [for review, see Ref. (33, 41)]. In mouse models to elicit antitumor immunity, immunotherapy using dual costimulatory agonist antibodies to CD134 (OX-40) and CD137 (4-1BB) induces Eomes in CD4 CTL that is necessary for GrB expression (42). Other groups have also demonstrated a role for Eomes in CD4 CTL differentiation after various immunotherapy regimens (43, 44); however, these therapies are designed to overcome tolerance induction and may reflect high levels of activation in the CD4 T cell compartment, driving toward a more CD8-like phenotype. In contrast, during IAV infection, Eomes expression is low in CD4 CTL in the lung (20) and does not appear to be regulated by IL-2 signaling (22). Conversely, IAV-specific CD4 CTL in the lung express more T-bet than cells in the DLN and spleen, and this expression is regulated by IL-2 and IFN-α *in vivo* (20–22). When T-bet is knocked out, CD4 T cells in the lung express less GrB and perforin, but addition of T-bet to CD4 T cells from SLO does not increase GrB expression, indicating that T-bet alone is not sufficient to induce CD4 CTL (21). The transcription factor shown to regulate both T-bet and cytolytic proteins in CD4 T cells is Blimp-1, a transcriptional repressor. Blocking IFN-α signaling was shown to decrease Blimp-1 expression in CD4 CTL in the lungs of IAV-infected mice. Moreover, Blimp-1 was required for CD4 cell expression of GrB and cytolytic activity and protection against IAV infection *in vivo* (21). Blimp-1 was also shown to promote T-bet binding to GrB and Prf1 promotor regions, thus regulating cytolytic activity at the molecular level. Comparisons between the tumor immunotherapy and infection models reveal that different stimuli can induce distinct transcription factor expression in CD4 CTL with dual costimulation inducing Eomes and IAV infection inducing T-bet and Blimp-1. However, until the transcription factors involved in CD4 CTL development become more clearly defined, the debate whether CD4 CTL make up a distinct subset of the CD4 T cell lineage or are a terminally differentiated Th1 cell will remain unresolved.

## Are CD4 CTL Present in the Memory Pool?

It is clear that CD4 CTL arise during IAV infection and both cytokines and transcription factors are important in driving CD4 CTL differentiation. However, questions still remain regarding the memory potential of these cells and whether they transition to CD4 memory cell populations resident in the lung or SLO. Studies in humans suggest that CD4 CTL are present in the memory pool, since these effectors are described as “preexisting” in experimental IAV infection (19) and in vaccinated older adults (37). However, in IAV vaccination studies in humans (37) and in mouse models of vaccination (45) or secondary infection (46), the cells that are being assayed for are probably more aptly named secondary effectors. CD4 CTL, such as CD8 memory CTL, at the memory stage do not express cytolytic proteins until restimulated *in vitro* (37, 45, 47) and without identification of cell surface markers, the transition from effector CD4 CTL to memory is difficult to monitor. Just recently, the class I-restricted T cell-associated molecule (CRTAM) known to be expressed on CD8 T cells and NK cells has been identified on a fraction of CD4 T cells that develop cytotoxicity *in vivo* (39). CRTAM^+^ CD4 CTL can be isolated from the lung after IAV infection and lyse NP peptide pulsed targets, while CD4 cells from CRTAM-deficient mice are not cytotoxic. CRTAM interacts with its ligand CADM1, expressed on epithelial cells and CD8α^+^ DC, and there is speculation that CRTAM^+^ CD4 CTL interact with this ligand to expand memory phenotype cells *in vivo* (39). Whether CRTAM^+^ CD4 cells can be identified in the memory pool and whether these cells become secondary effectors capable of cytolytic activity in the lung are questions that remain to be answered. Another cell surface receptor that may be important in identifying CD4 CTL is the NK cell lectin receptor NKG2A. Workman et al. have demonstrated that a proportion of CD4^+^GrB^+^ cells in the lung at the effector stage express NKG2A/C/E using an antibody that can recognize all three isoforms (22, 40). Moreover, NKG2A gene expression was apparent in CD4 Th1 memory cell clones after IAV infection (48), and NKG2A protein expression was higher in secondary effectors found in the lung, compared to secondary effectors in the DLN (46). Interestingly, Eomes expression was also increased in secondary effectors suggesting Eomes may be more important for driving memory or secondary CD4 CTL responses (46, 49), but not effector CD4 CTL responses, similar to studies with CD8 CTL memory (41). Kinetic studies investigating the expression patterns of CRTAM and NKG2A and their correlation with degranulation on CD4 CTL over time may yield valuable information regarding the transition from effector to memory CD4 CTL. This would be a breakthrough in the study of CD4 CTL as currently there exists no clear cell surface marker that identifies this cell type. This would also allow a more detailed analysis of the role of Blimp-1 and Eomes in inducing GrB and perforin re-expression in memory CD4 CTL.

Experiments to dissect the role of transcription factors important in innate cell signaling during IAV infection are on going and have demonstrated that interferon response factor-3 (IRF-3) regulates GrB expression in memory CD4 and CD8 CTL (47). Interestingly, IRF-3 does not appear to play a role in driving GrB expression at the effector stage after IAV infection, but has marked effects on antigen-specific GrB re-expression in the memory CD8 population. This defect was most apparent in the central memory phenotype and was not abrogated by innate cytokines, such as IL-12, IFN-β, or IL-15 (47). The level of GrB re-expression in memory CD4 CTL, especially in the SLO, is difficult to detect; however, it is thought the same defect exists in CD4 CTL (47). Studies are underway to fully understand the relationship between IRF3 and GrB expression and whether IRF3 functions intrinsically in T cells or whether IRF3-controlled cytokines ultimately drive GrB expression in the memory phase.

## Can Vaccination Induce CD4 CTL for Protection against IAV?

As discussed above, immunotherapy or vaccination to elicit antitumor immunity results in CD4 CTL poised to eradicate established tumors, yet these strategies use agonistic antibodies to costimulatory molecules to induce high levels of activation and overcome tolerogenic signals (42–44). Recently, there have been two reports that demonstrate vaccine induced CD4 CTL generation *via* TLR4 agonist and protein (50) or complete Freund’s adjuvant (CFA) and viral MCMV peptide (18) immunization. However, the first study demonstrated that CD4 CTL generated *via* TLR4 agonist administration expressed granzyme A and killed *via* CD40:CD40L interactions with no role for GrB, perforin, or FasL-mediated cell death (50). The second study vaccinated mice against murine CMV using peptide and CFA immunization and found that CD4 CTL expressing GrB mediated viral clearance upon challenge; however, the mechanism of cell killing by vaccine-induced CD4 CTL was not elucidated (18). Thus, determining the role of vaccine induced CD4 CTL in protection against infections is the next step in this area of intense research.

One of the more important studies of vaccination-induced CD4 CTL generation comes from work by Zhou and McElhaney investigating the role of IAV vaccine-induced protection in elderly populations (36, 37, 51). It is well documented that elderly subjects have diminished immune responses to vaccination with B cell, helper T cell, and cytotoxic T cell responses all blunted to some degree (52). Surprisingly, while antibody responses did not correlate to protection from IAV infection in elderly patients, the existence of vaccine-induced GrB^+^ cells and the level of GrB expression correlated to a decreased risk for influenza illness (36). Moreover, healthy older adults showed GrB and perforin expression in both the CD4 and CD8 compartments at 4 weeks post-vaccination, albeit, lower than in healthy young adults. Interestingly, at 10 weeks post-vaccination, cytotoxicity in the CD8 compartment decreased dramatically in older adults compared to young adults, while cytotoxicity in the CD4 compartment was not significantly decreased (37). These experiments suggest that the CD4 CTL population in adults, especially the elderly, can be exploited by more universal vaccines that included conserved, internal T cell epitopes and promote cell-mediated immune responses.

Presently, work by Vogel et al. is aimed at enhancing current IAV vaccine strategies by incorporating pattern-recognition receptor (PRR) agonists as adjuvants and by delivering inactivated virus and adjuvant directly to the respiratory mucosa by intranasal immunization. To this end, single-dose intranasal administration of TLR-9 agonist, CpG, and heat-inactivated IAV was shown to promote heterosubtypic protection against lethal IAV challenge as measured by reduced viral titers (45). In addition, GrB expressing CD4 effectors were present in the lung after priming with CpG and inactivated IAV, as well as after heterosubtypic challenge 28 days later. Interestingly, low dose x31 (H3N2) immunization did not induce GrB expressing CD4 effectors at the primary response, but did generate high numbers of CD4 CTL in the secondary response (45), again suggesting that the inflammatory microenvironment in the lung may provide second signals for complete differentiation of CD4 CTL, as x31 infection does not promote as high of an inflammatory response compared to CpG (45) or PR8 (40). Studies are underway to definitively demonstrate the cytolytic capacity of vaccine-induced CD4 CTL in our model and to determine if other PRR agonist combinations can induce the CD4 CTL phenotype in mucosal tissues.

## Concluding Remarks

Taken together, CD4 CTL are an important component of the cell-mediated immune response against IAV infection. IAV-specific CD4 CTL are induced *via* IL-2 and inflammatory cytokine signaling *in vivo* and appear to require the transcription factor Blimp-1 for development. Phenotypically, CD4 CTL resemble Th1 effector cells that also express GrB and perforin and have direct cytotoxic activity *ex vivo*. CD4 CTL show perforin-dependent protection against lethal IAV infection in mouse models of infection, as well as in human studies of experimental infection or vaccination. There remains more to discover regarding this potentially unique subset of CD4 T cells. The identification of cell surface markers, such as CRTAM and NKG2A, will allow a more detailed analysis of the memory and secondary effector potential of these cells. These insights will provide a greater understanding of CD4 CTL and information for the development of vaccine strategies that induce an extra armament of heterosubtypic protection against IAV strains with pandemic potential.

## Author Contributions

DB, AL, and AW organized, wrote, and edited the manuscript.

## Conflict of Interest Statement

The authors declare that the research was conducted in the absence of any commercial or financial relationships that could be construed as a potential conflict of interest.

## Abbreviations

CD4 CTL, CD4 T cell with cytolytic potential; CTL, cytotoxic T lymphocyte; DLN, draining lymph node; GrB, granzyme B; IAV, influenza A virus; PR8, influenza A/Puerto Rico/8/34; Prf1, perforin; SLO, secondary lymphoid organs.

## References

[1] Swain SL McKinstry KK Strutt TM Expanding roles for CD4(+) T cells in immunity to viruses. Nat Rev Immunol (2012) 12:136–48. 10.1038/nri3152 22266691 PMC3764486

[2] Zhu J Yamane H Paul WE. Differentiation of effector CD4 T cell populations (*). Annu Rev Immunol (2010) 28:445–89. 10.1146/annurev-immunol-030409-101212 20192806 PMC3502616

[3] Dennert G Swain SL Waterfield JD Warner JF Dutton RW. Fine specificity mapping of two allospecific T cell lines: recognition of private specificities in the H-2 IA subregion. Eur J Immunol (1981) 11:62–4. 10.1002/eji.1830110113 6163637

[4] Swain SL Dennert G Wormsley S Dutton RW. The Lyt phenotype of a long-term allospecific T cell line. Both helper and killer activities to IA are mediated by Ly-1 cells. Eur J Immunol (1981) 11:175–80. 10.1002/eji.1830110304 6972304

[5] Lukacher AE Morrison LA Braciale VL Malissen B Braciale TJ. Expression of specific cytolytic activity by H-2I region-restricted, influenza virus-specific T lymphocyte clones. J Exp Med (1985) 162:171–87. 10.1084/jem.162.1.171 2409206 PMC2187708

[6] Appay V The physiological role of cytotoxic CD4(+) T-cells: the holy grail? Clin Exp Immunol (2004) 138:10–3. 10.1111/j.1365-2249.2004.02605.x 15373899 PMC1809194

[7] Appay V Zaunders JJ Papagno L Sutton J Jaramillo A Waters A Characterization of CD4(+) CTLs ex vivo. J Immunol (2002) 168:5954–8. 10.4049/jimmunol.168.11.5954 12023402

[8] Casazza JP Betts MR Price DA Precopio ML Ruff LE Brenchley JM Acquisition of direct antiviral effector functions by CMV-specific CD4+ T lymphocytes with cellular maturation. J Exp Med (2006) 203:2865–77. 10.1084/jem.20052246 17158960 PMC2118179

[9] Soghoian DZ Jessen H Flanders M Sierra-Davidson K Cutler S Pertel T HIV-specific cytolytic CD4 T cell responses during acute HIV infection predict disease outcome. Sci Transl Med (2012) 4:123ra125. 10.1126/scitranslmed.3003165 22378925 PMC3918726

[10] Soghoian DZ Streeck H. Cytolytic CD4(+) T cells in viral immunity. Expert Rev Vaccines (2010) 9:1453–63. 10.1586/erv.10.132 21105780 PMC3033049

[11] Heller KN Gurer C Munz C. Virus-specific CD4+ T cells: ready for direct attack. J Exp Med (2006) 203:805–8. 10.1084/jem.20060215 16549599 PMC2118265

[12] Paludan C Bickham K Nikiforow S Tsang ML Goodman K Hanekom WA Epstein-Barr nuclear antigen 1-specific CD4(+) Th1 cells kill Burkitt’s lymphoma cells. J Immunol (2002) 169:1593–603. 10.4049/jimmunol.169.3.1593 12133989

[13] Jellison ER Kim SK Welsh RM. Cutting edge: MHC class II-restricted killing in vivo during viral infection. J Immunol (2005) 174:614–8. 10.4049/jimmunol.174.2.614 15634878

[14] Zajac AJ Quinn DG Cohen PL Frelinger JA. Fas-dependent CD4+ cytotoxic T-cell-mediated pathogenesis during virus infection. Proc Natl Acad Sci U S A (1996) 93:14730–5. 10.1073/pnas.93.25.14730 8962123 PMC26204

[15] Stuller KA Cush SS Flano E. Persistent gamma-herpesvirus infection induces a CD4 T cell response containing functionally distinct effector populations. J Immunol (2010) 184:3850–6. 10.4049/jimmunol.0902935 20208003 PMC3044337

[16] Stuller KA Flano E. CD4 T cells mediate killing during persistent gammaherpesvirus 68 infection. J Virol (2009) 83:4700–3. 10.1128/JVI.02240-08 19244319 PMC2668489

[17] Fang M Siciliano NA Hersperger AR Roscoe F Hu A Ma X Perforin-dependent CD4+ T-cell cytotoxicity contributes to control a murine poxvirus infection. Proc Natl Acad Sci U S A (2012) 109:9983–8. 10.1073/pnas.1202143109 22665800 PMC3382508

[18] Verma S Weiskopf D Gupta A McDonald B Peters B Sette A Cytomegalovirus-specific CD4 T cells are cytolytic and mediate vaccine protection. J Virol (2015) 90:650–8. 10.1128/JVI.02123-15 26491148 PMC4702662

[19] Wilkinson TM Li CK Chui CS Huang AK Perkins M Liebner JC Preexisting influenza-specific CD4+ T cells correlate with disease protection against influenza challenge in humans. Nat Med (2012) 18:274–80. 10.1038/nm.2612 22286307

[20] Brown DM Lee S Garcia-Hernandez Mde L Swain SL. Multifunctional CD4 cells expressing gamma interferon and perforin mediate protection against lethal influenza virus infection. J Virol (2012) 86:6792–803. 10.1128/JVI.07172-11 22491469 PMC3393557

[21] Hua L Yao S Pham D Jiang L Wright J Sawant D Cytokine-dependent induction of CD4+ T cells with cytotoxic potential during influenza virus infection. J Virol (2013) 87:11884–93. 10.1128/JVI.01461-13 23986597 PMC3807312

[22] Workman AM Jacobs AK Vogel AJ Condon S Brown DM. Inflammation enhances IL-2 driven differentiation of cytolytic CD4 T cells. PLoS One (2014) 9:e89010. 10.1371/journal.pone.0089010 24586481 PMC3930678

[23] Stalder T Hahn S Erb P. Fas antigen is the major target molecule for CD4+ T cell-mediated cytotoxicity. J Immunol (1994) 152:1127–33. 7507960

[24] Brown DM. Cytolytic CD4 cells: direct mediators in infectious disease and malignancy. Cell Immunol (2010) 262:89–95. 10.1016/j.cellimm.2010.02.008 20236628 PMC2874968

[25] Graham MB Braciale VL Braciale TJ. Influenza virus-specific CD4+ T helper type 2 T lymphocytes do not promote recovery from experimental virus infection. J Exp Med (1994) 180:1273–82. 10.1084/jem.180.4.1273 7931062 PMC2191682

[26] Brown DM Dilzer AM Meents DL Swain SL. CD4 T cell-mediated protection from lethal influenza: perforin and antibody-mediated mechanisms give a one-two punch. J Immunol (2006) 177:2888–98. 10.4049/jimmunol.177.5.2888 16920924

[27] Brown DM Kamperschroer C Dilzer AM Roberts DM Swain SL. IL-2 and antigen dose differentially regulate perforin- and FasL-mediated cytolytic activity in antigen specific CD4+ T cells. Cell Immunol (2009) 257:69–79. 10.1016/j.cellimm.2009.03.002 19338979 PMC2683476

[28] McKinstry KK Strutt TM Kuang Y Brown DM Sell S Dutton RW Memory CD4+ T cells protect against influenza through multiple synergizing mechanisms. J Clin Invest (2012) 122:2847–56. 10.1172/JCI63689 22820287 PMC3408751

[29] Teijaro JR Verhoeven D Page CA Turner D Farber DL. Memory CD4 T cells direct protective responses to influenza virus in the lungs through helper-independent mechanisms. J Virol (2010) 84:9217–26. 10.1128/JVI.01069-10 20592069 PMC2937635

[30] McKinstry KK Dutton RW Swain SL Strutt TM. Memory CD4 T ­cell-mediated immunity against influenza A virus: more than a little helpful. Arch Immunol Ther Exp (Warsz) (2013) 61:341–53. 10.1007/s00005-013-0236-z 23708562 PMC3874125

[31] Hildemann SK Eberlein J Davenport B Nguyen TT Victorino F Homann D. High efficiency of antiviral CD4(+) killer T cells. PLoS One (2013) 8:e60420. 10.1371/journal.pone.0060420 23565245 PMC3614903

[32] Brennan AJ House IG Oliaro J Ramsbottom KM Hagn M Yagita H A method for detecting intracellular perforin in mouse lymphocytes. J Immunol (2014) 193:5744–50. 10.4049/jimmunol.1402207 25348626

[33] Pipkin ME Rao A Lichtenheld MG. The transcriptional control of the perforin locus. Immunol Rev (2010) 235:55–72. 10.1111/j.0105-2896.2010.00905.x 20536555 PMC4749266

[34] Pipkin ME Sacks JA Cruz-Guilloty F Lichtenheld MG Bevan MJ Rao A. Interleukin-2 and inflammation induce distinct transcriptional programs that promote the differentiation of effector cytolytic T cells. Immunity (2010) 32:79–90. 10.1016/j.immuni.2009.11.012 20096607 PMC2906224

[35] Lin L Couturier J Yu X Medina MA Kozinetz CA Lewis DE. Granzyme B secretion by human memory CD4 T cells is less strictly regulated compared to memory CD8 T cells. BMC Immunol (2014) 15:36. 10.1186/s12865-014-0036-1 25245659 PMC4195902

[36] McElhaney JE Ewen C Zhou X Kane KP Xie D Hager WD Granzyme B: correlates with protection and enhanced CTL response to influenza vaccination in older adults. Vaccine (2009) 27:2418–25. 10.1016/j.vaccine.2009.01.136 19368783 PMC2800816

[37] Zhou X McElhaney JE. Age-related changes in memory and effector T cells responding to influenza A/H3N2 and pandemic A/H1N1 strains in humans. Vaccine (2011) 29:2169–77. 10.1016/j.vaccine.2010.12.029 21353149 PMC3057414

[38] Sridhar S Begom S Bermingham A Hoschler K Adamson W Carman W Cellular immune correlates of protection against symptomatic pandemic influenza. Nat Med (2013) 19:1305–12. 10.1038/nm.3350 24056771

[39] Takeuchi A Badr ME Miyauchi K Ishihara C Onishi R Guo Z CRTAM determines the CD4+ cytotoxic T lymphocyte lineage. J Exp Med (2015) 213: 123–38. 10.1084/jem.20150519 26694968 PMC4710199

[40] Brown DM Workman AM Vogel AJ Condon S Inflammation regulates the differentiation of CD4 T cells with cytolytic potential. J Immunol (2013) 190:173.171.

[41] Kaech SM Cui W. Transcriptional control of effector and memory CD8+ T cell differentiation. Nat Rev Immunol (2012) 12:749–61. 10.1038/nri3307 23080391 PMC4137483

[42] Qui HZ Hagymasi AT Bandyopadhyay S St Rose MC Ramanarasimhaiah R Menoret A CD134 plus CD137 dual costimulation induces Eomesodermin in CD4 T cells to program cytotoxic Th1 differentiation. J Immunol (2011) 187:3555–64. 10.4049/jimmunol.1101244 21880986 PMC3178659

[43] Curran MA Geiger TL Montalvo W Kim M Reiner SL Al-Shamkhani A Systemic 4-1BB activation induces a novel T cell phenotype driven by high expression of Eomesodermin. J Exp Med (2013) 210:743–55. 10.1084/jem.20121190 23547098 PMC3620352

[44] Hirschhorn-Cymerman D Budhu S Kitano S Liu C Zhao F Zhong H Induction of tumoricidal function in CD4+ T cells is associated with concomitant memory and terminally differentiated phenotype. J Exp Med (2012) 209:2113–26. 10.1084/jem.20120532 23008334 PMC3478933

[45] Vogel AJ Brown DM. Single-dose CpG immunization protects against a heterosubtypic challenge and generates antigen-specific memory T cells. Front Immunol (2015) 6:327. 10.3389/fimmu.2015.00327 26161083 PMC4479795

[46] Strutt TM McKinstry KK Kuang Y Bradley LM Swain SL. Memory CD4+ T-cell-mediated protection depends on secondary effectors that are distinct from and superior to primary effectors. Proc Natl Acad Sci U S A (2012) 109:E2551–60. 10.1073/pnas.1205894109 22927425 PMC3458385

[47] Moore TC Vogel AJ Petro TM Brown DM. IRF3 deficiency impacts granzyme B expression and maintenance of memory T cell function in response to viral infection. Microbes Infect (2015) 17:426–39. 10.1016/j.micinf.2015.03.001 25777301 PMC4479197

[48] Graham CM Christensen JR Thomas DB. Differential induction of CD94 and NKG2 in CD4 helper T cells. A consequence of influenza virus infection and interferon-gamma? Immunology (2007) 121:238–47. 10.1111/j.1365-2567.2007.02563.x 17462078 PMC2265943

[49] Strutt TM McKinstry KK Marshall NB Vong AM Dutton RW Swain SL. Multipronged CD4(+) T-cell effector and memory responses cooperate to provide potent immunity against respiratory virus. Immunol Rev (2013) 255:149–64. 10.1111/imr.12088 23947353 PMC4206082

[50] Coler RN Hudson T Hughes S Huang PW Beebe EA Orr MT. Vaccination produces CD4 T cells with a novel CD154-CD40-dependent cytolytic mechanism. J Immunol (2015) 195:3190–7. 10.4049/jimmunol.1501118 26297758 PMC4575887

[51] McElhaney JE Coler RN Baldwin SL. Immunologic correlates of protection and potential role for adjuvants to improve influenza vaccines in older adults. Expert Rev Vaccines (2013) 12:759–66. 10.1586/14760584.2013.811193 23885821

[52] Haq K McElhaney JE. Immunosenescence: influenza vaccination and the elderly. Curr Opin Immunol (2014) 29:38–42. 10.1016/j.coi.2014.03.008 24769424

